# Factors affecting unmet healthcare needs of low-income overweight and obese women in Korea: analysis of the Korean National Health and Nutrition Examination Survey 2017

**DOI:** 10.4069/kjwhn.2021.05.06

**Published:** 2021-06-17

**Authors:** Ju-Hee Nho, Sook Kyoung Park

**Affiliations:** College of Nursing, Jeonbuk Research Institute of Nursing Science, Jeonbuk National University, Jeonju, Korea

**Keywords:** Health services needs and demand, Income, Obesity, Overweight, Women

## Abstract

**Purpose:**

The purpose of this study was to explore unmet healthcare needs among low-income overweight and obese women and to identify the factors affecting unmet healthcare needs.

**Methods:**

The study was a secondary analysis of data from the 2017 Korea National Health and Nutrition Examination Survey. A final sample of 388 out of 8,127 participants was analyzed using complex descriptive statistics, the chi-square test, the independent t-test, and logistic regression.

**Results:**

The mean age of the participants was 66.51±1.05 years. Unmet healthcare needs were experienced by 19.4% of low-income overweight and obese women. Women with depression, stress, and poor self-reported health status were significantly more likely than their counterparts to experience unmet healthcare needs. Poor self-reported health status was confirmed to be related to unmet health needs in low-income overweight and obese women (odds ratio, 2.65; *p*=.011).

**Conclusion:**

The study provides the novel insight that the unmet healthcare needs of low-income overweight and obese women were influenced by self-reported health status. Healthcare providers should make efforts to develop strategies to reduce unmet healthcare needs among low-income overweight and obese women, who constitute a vulnerable population.

## Introduction

The prevalence of obesity among adults over the age of 19 in South Korea (hereafter, Korea) increased by 2.5% points from 31.9% in 2009 to 33.4% in 2015 and further increased to 34.4% in 2019. Classified by income level, the prevalence of obesity among middle and upper-income earners was 33.7%, whereas it was 34.5% among middle and lower-income earners and 35.6% among lower-income earners. Disparities were also seen according to the residential area, as the prevalence of obesity was 33.6% among those living in urban areas and 38.3% for residents of rural areas, which have lower income levels; overall, the prevalence of obesity was higher among those living in rural settings [1]. Among women in particular, an interaction was observed between the effects of income and residential area. The overall obesity rate of higher-income women was 7% points higher than that of lower-income women, but this disparity was wider (9.3% points) among those living in rural areas. Furthermore, the obesity rate among women with a low level of education was 6.3% points higher than that among women with a high level of education [1]. These observations indicate that obesity in Korean women is associated with low socioeconomic status, as has also been observed in other developed countries.

As expressed by the saying that “obesity is a disease” [2], obesity is associated with endometrial cancer and breast cancer in addition to chronic diseases commonly encountered in adulthood, such as diabetes and hypertension [3]. Furthermore, 25% of obese women experienced depression [4]. Obesity increases the risk of anovulation, menstrual irregularities, polycystic ovarian syndrome, and impaired endometrial receptivity and implantation, and obese women were found to be more than three times more likely to become infertile than normal-weight women [3]. Of particular note, it has been reported that low-income women ate larger quantities of food with a high glycemic index and lipid content [5]. Compounded by a lack of conditions for exercise and high treatment costs making medical access less accessible, low-income women are more likely to become obese [6]. It has been found that among low-income women, those with obesity had a lower quality of life than normal-weight women, and their health status was perceived somewhat negatively [7]. Attention is needed to alleviate the health inequalities caused by income and gender gaps.

Unmet healthcare needs refer to a situation where adequate prevention, alleviation, and treatment of a disease or disability cannot be provided due to a lack of medical services [8]. The presence of unmet healthcare needs increases disease severity and increases the likelihood of complications and mortality [9]. In particular, unmet healthcare needs due to socioeconomic problems have a negative effect on psychological factors; for example, they increase social isolation and depression by causing relative deprivation [10]. Social isolation and depression, in turn, cause suicidal thoughts [11] and significantly lower individuals’ quality of life. Unmet healthcare needs were found to be associated with low income, low education level, chronic illness, living alone, and limited activities [12,13]. Low income has also been found to be related to poor health status as well as unmet healthcare needs [14,15]. In a study of Korean adults based on data from the Korean Health Panel, it was reported that economic reasons had a mediating effect of 14.7% to 32.9% of unmet healthcare needs [16]. Of note, income had a stronger effect on unmet healthcare needs among women than among men [17]. As described above, overweight and obese women in low-income groups have specific health care needs, so it is necessary to understand the status of their unmet healthcare needs and the factors affecting them. With increasing interest in unmet healthcare needs, factors related to unsatisfactory medical care for adults, married migrant women, the elderly, and single-person households have been studied [12-18]. However, research has yet to explore the status of unmet healthcare needs and related factors among low-income women who are overweight and obese in Korea, underscoring the need for efforts to improve health by identifying and improving the factors associated with the health inequalities that these women experience.

Anderson’s behavioral model was developed for the identification and evaluation of factors related to medical service use, and it is a useful model for identifying social and personal determinants of medical service use [19]. Anderson’s behavioral model has been used not only to analyze medical service use behavior but also to identify systematic factors influencing unmet healthcare needs in research aiming to achieve equitable access to medical service use [18,20]. Therefore, in this study, the classification in Anderson’s behavioral model (predisposing, enabling, and need factors) was used to explore the factors influencing unmet healthcare needs. Predisposing factors are characteristics that an individual already has before the occurrence of medical needs, and include demographic factors such as age, sex, and education. Enabling factors include economic and sociological factors such as income level, employment, family resources, and insurance as factors that enable medical services to be used, and need factors are related to the level of an individual’s disability or disease, including overall health status and symptoms [19].

As previously discussed, low-income women who are overweight and obese experience a variety of health problems. Therefore, this study identified aspects of their individual, demographic, social, and economic vulnerabilities and unmet healthcare needs. In light of the need to determine the factors that affect unmet healthcare needs, the present study, based on Anderson’s behavioral model, aimed to identify the factors influencing unmet healthcare needs among low-income women who are overweight and obese. It is expected that these findings will inform a personal and socioeconomic approach to improve the health of these women in the future.

### Objectives

The purpose of this study was to obtain insights into the unmet healthcare needs of low-income women who are overweight and obese in Korea, and to identify the factors influencing unmet healthcare needs based on Anderson’s behavioral model of medical care. The specific aims were as follows:

First, to investigate the status of unmet healthcare needs for low-income women who are overweight and obese.

Second, to identify differences in unmet healthcare needs according to predisposing factors, enabling factors, and need factors of low-income women who are overweight and obese.

Third, to analyze factors influencing unmet healthcare needs of low-income women who are overweight and obese.

## Methods

Ethics statement: This study was exempted by the Institutional Review Board (IRB) of Jeonbuk National University (2020-08-018), as this was secondary analysis of existing data and the data were handled anonymously.

### Study design

This study was conducted to obtain insights into the characteristics of unmet healthcare needs and factors influencing those needs among overweight and obese low-income women. This secondary analysis used health survey data from the seventh Korea National Health and Nutrition Examination Survey (KNHANES) (2017) and analyzed the data based on Anderson’s behavioral model of medical care. This study was described in accordance with the Strengthening the Reporting of Observational Studies in Epidemiology (STROBE) guidelines (https://www.strobe-statement.org/index.php?id=strobe-home).

### Data sources

**Figure 1.**
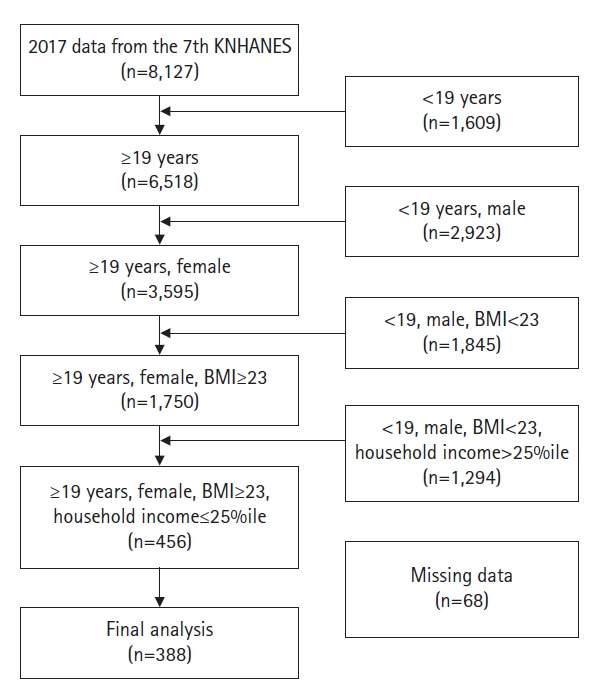
Flowchart of the study population. BMI: Body mass index (kg/m^2^); KNHANES: Korea National Health and Nutrition Examination Survey.

This study used data from the seventh wave of the KNHANES, which is conducted annually, with the approval of the Research Ethics Review Committee from the Korea Centers for Disease Control and Prevention. Only the 2017 KNHANES contained a survey on whether participants had experienced “depression for 2 weeks in a row”; therefore, this study used data from 2017. From the total number of participants in the 2017 data from the 7th KNHANES, 6,518 adult women aged 19 years or older were extracted. Using a body mass index of 23 kg/m^2^ as the cutoff [21], there were 456 overweight or obese women whose household income was in the lowest quartile. Sixty-eight participants were excluded if there was no information or answers of “don’t know” for relevant variables (marital status, 1; education, 38; economic activity, 38; type of health insurance, 18; whether they subscribed to private insurance, 5; diabetes diagnosis, 1; tuberculosis diagnosis, 32; cancer diagnosis, 32; aerobic physical activity, 41; whether they have felt depressed for at least 2 weeks, 14; perceived stress, 14; and subjective health status, 31). In total, data from 388 respondents were extracted and analyzed (Figure 1).

### Study variables

#### Unmet healthcare needs

Medical use data from the KNHANES were used to assess unmet healthcare needs, which were defined as an answer of “yes” to the item asking, “did you ever need treatment (examination or treatment) in a hospital (excluding dentistry) in the last year?”

#### Low income

Low-income status was defined based on the household income quartile, which is a basic variable in the KNHANES. Household income was classified into lower, middle-low, middle-high, and upper quartiles, and those who fell into the lower quartile were defined as low income.

#### Predisposing factors

The predisposing factors included age, marital status, education level, and the number of family members living together. Age was classified into under 60 years, 60 to 79 years, and 70 years or older, and marital status was divided into married, bereaved, divorced, and single. Education level was classified as elementary school or lower, middle school, high school, and college or higher. In addition, the number of family members living together was reclassified to divide respondents into those who lived alone and those who lived with at least one other family member.

#### Enabling factors

Enabling factors were classified into employment, health insurance type, and private insurance coverage. Employment status was classified in terms of presence or absence, and the types of health insurance were classified as self-employed, employee, and dependent. Private insurance coverage was classified as “yes” or “no.”

#### Need factors

Need factors included hypertension, diabetes, and cancer, which are representative chronic diseases suggested by the Centers for Disease Control and Prevention [22], as well as tuberculosis, which has been identified as a relevant factor among socioeconomically vulnerable groups [23]. Aerobic physical activity, depression, stress, and perceived health status were analyzed. The presence of chronic diseases (hypertension, diabetes, tuberculosis, and cancer) was confirmed by doctors. Participants were classified as engaging or not engaging in aerobic physical activity, based on whether they took part in 2 hours and 30 minutes of moderate-intensity physical activity or 1 hour and 15 minutes of high-intensity physical activity per week, or a mixture of moderate and high-intensity physical activities (high-intensity of 1 minute=moderate-intensity of 2 minutes). Depression was classified as present or absent based on answers of “yes” and “no,” respectively, for the item asking whether participants had experienced feeling depressed for 2 weeks or more. According to previous studies [13,18], responses on perceived stress were dichotomized as “no” (responses of feeling less stress, feeling a little stress, or barely feeling stress in everyday life) or “yes” (feeling a lot of stress or feeling very much stress in everyday life). Perceived health status was classified as “very bad,” “bad,” “average,” “good,” or “very good”; these responses were merged into “poor” (very bad or bad), “moderate,” and “good” (good or very good).

### Data collection

This study was based on 2017 data from the seventh KNHANES. The KNHANES gathers information on the health level, health behavior, and food and nutrition intake of the Korean people, and involves a household member survey, a health survey, a medical examination, and a nutrition survey. The household member survey identifies the current status of households in a selected area and selects households to participate in the KNHANES. The health survey is divided into a household survey, a health interview survey, and a health behavior survey, and is conducted through an interview and a self-response survey. In addition, the medical examination includes physical measurements, blood pressure and pulse measurements, and blood and urine tests, and the nutritional survey gathers information on dietary behavior and food intake. The data are collected following a protocol developed by the Korea Centers for Disease Control and Prevention (now known as the Korea Disease Control and Prevention Agency) and analyzed according to the guidelines for use.

### Data analysis

Data analysis was conducted using IBM SPSS ver. 26.0 (IBM Corp., Armonk, NY, USA). According to the analysis guideline, the complex-sample design elements were reflected and analyzed with appropriate consideration of the sample.

1) The general characteristics of the respondents and the status of unmet healthcare needs were analyzed by a composite sample frequency analysis.

2) The differences in unmet healthcare needs according to the characteristics of the respondents were analyzed by the complex-sample t-test and the complex-sample Rao-Scott chi-square test.

3) Complex-sample multiple logistic regression analysis was performed for the factors influencing unmet healthcare needs.

## Results

### General characteristics of the respondents

There were a total of 388 respondents in this study, with an average age of 66.51±1.05 years. The majority (51.9%) were over 70 years of age. The most common marital status was married (47.7%), the most common education level was an elementary school (67.8%), and the average number of family members living together was 2.04±0.07, while 33.5% of participants lived alone. Slightly more than two-thirds of the respondents (67.7%) were not employed, while 49.4% had employee-based national health insurance coverage and 59.1% of them did not have private insurance.

Hypertension was the most common chronic disease (52.2%), followed in order by diabetes (21.5%), cancer (8.3%), and tuberculosis (3.5%). Slightly more than three-quarters of respondents (75.6%) did not engage in aerobic physical activity, 30.4% had depression, and 34.3% perceived stress in their daily lives. The most common perceived health status was moderate (49.2%), followed by poor (42.9%) and good (7.9%) (Table 1).

### Unmet healthcare needs

Among the respondents of this study, 74 (19.4%) had unmet healthcare needs, while 314 (80.6%) had not experienced unmet healthcare needs (Table 2).

### Unmet healthcare needs according to respondents’ characteristics

Table 3 shows the differences in unmet healthcare needs according to the general characteristics of the respondents of this study. No significant differences were found in predisposing factors or enabling factors according to unmet healthcare needs. Among the need factors, the presence of depression was significantly associated with unmet healthcare needs (F=4.27, *p*=.041), as was perceived stress (F=5.93, *p*=.017). Unmet healthcare needs were also more common in respondents with poor perceived health (F=6.92, *p*=.001).

### Factors influencing unmet healthcare needs in low-income women who are overweight and obese

Among the need factors, perceived health status was identified as a factor affecting unmet healthcare needs among the respondents of this study. The odds of having unmet healthcare needs were approximately 60% lower in those with moderate self-reported health than in those with poor self-reported health (odds ratio, 0.40; 95% confidence interval, 0.20–0.78; *p*=.007) (Table 4).

## Discussion

This study investigated the status of unmet healthcare needs and identified factors influencing unmet healthcare needs among low-income women who were overweight and obese based on 2017 data from the seventh KNHANES. The factors influencing unmet healthcare needs were categorized into predisposing factors, enabling factors, and need factors according to Anderson’s model of medical use behavior. It is expected that the results will be used as basic data for the preparation of alternative strategies to reduce unmet healthcare needs and the development of health promotion programs for this population.

It was found that 75.6% of low-income women who were overweight and obese did not engage in aerobic physical activity, 30.4% had depression, and 34.3% perceived stress in their everyday lives, Only 7.9% of respondents reported good perceived health. In the 2018 KNHANES results, it was reported that 47.6% of adults over 19 years old in Korea engaged in aerobic physical activity, including 38.9% of women [24]. The corresponding proportion in this study was much lower. Exercise and lifestyle intervention programs have been shown to be effective in promoting physical activity in overweight and obese women [25,26]. Interventions should be considered to promote physical activity for low-income women. In addition, the proportions of respondents reporting depression and perceived stress were higher than those of Korean adult women overall (13.7% and 28.6%, respectively) [27]. Likewise, the proportion of respondents with good perceived health was lower than that (23.2%) among adult women over 20 years of age based on 2020 data from Statistics Korea [28]. These results show that low-income women who are overweight and obese have relatively poor physical and psychological health, underscoring the need to pursue personal and social structural strategies to improve their physical and psychological health.

The frequency of unmet healthcare needs among low-income women who were overweight and obese was 19.4%. This is higher than the result of a survey of 134,072 people based on the results of the Canadian community health survey, which showed that the frequency of unmet healthcare needs among adult women was 12.6% [20], and a corresponding rate of only 1.6% was reported in Thailand [29]. Country-specific differences may result from differences in medical service systems and insurance systems from country to country; nonetheless, the proportion of women with unmet healthcare needs observed in the present study can be seen as quite high. For instance, the frequency of unmet healthcare needs found in the present study is higher than that (17.3%) reported in a previous study of women in single households using 2017 KNHANES data [13] and the rate (15.75%) reported among adults in Korea using panel data [30]. In addition, the majority of respondents in this study were in their 70s or older, had a low education level, and were not engaged in economic activities, and 33.5% of them lived alone. Among all overweight and obese adult women in the KNHANES data, 25% were low income (in the lowest income quartile), and many did not have private insurance. These results show the need for an approach to resolve health inequality among adults older than 60 who live alone. In particular, women had a 3.8% points higher likelihood of having unmet healthcare needs than men who lived alone [13]. Low-income women were found to experience frequent difficulties accessing medical services and communicating with medical staff [31]. To develop measures to reduce unmet healthcare needs, there is a need to develop a policy that takes into account these women’s characteristics (i.e., a gender-sensitive policy). In addition, a more systematic approach and efforts to provide sufficient medical services based on a detailed assessment of these women’s needs and problems are necessary.

This study found that depression, perceived stress, and poor perceived health status were associated with a significantly higher likelihood of unmet healthcare needs. This is consistent with the results of previous studies [32,33], indicating that unmet healthcare needs are more common in adults over 19 years of age, including the elderly, who have depression, stress, and poor perceived health status. It has been reported that stress reduces or eliminates individuals’ will to use medical services, causing frustration and resulting in unmet healthcare needs [34]. In addition, depressed individuals pay less attention to the positives and focus on the negatives [35,36], meaning that even if their needs for treatment are satisfied, they may not perceive the situation accordingly; their continuing perception of being “unsatisfied” may itself act as a risk factor for avoiding treatment [37-39], resulting in unmet healthcare needs. Therefore, it is necessary to develop strategies to reduce unmet healthcare needs through interventions and approaches that can assess mental health status and reduce depression and stress.

In this study, the differences in unmet healthcare needs and factors influencing unmet healthcare needs were examined according to predisposing, enabling, and need factors based on Anderson’s model of medical use behavior. Perceived health status was investigated as a need factor, and it was found that moderate perceived health status was associated with a 60.4% lower chance of having unmet healthcare needs. This is similar to the results of previous studies [40-42], which found that the subjective health status of moderate or poor was associated with unmet healthcare needs. Based on these results, it was suggested that efforts to reduce unmet healthcare needs should focus on individuals who perceive their health status as poor [13]. In this study, health status perceived at moderate level was associated with a lower likelihood of unmet healthcare needs. A study of 11,378 adults in Korea found that the risk of unmet healthcare needs was 1.46 times higher for those with moderate perceived health status than in those who perceived their health status as good [39], showing the need to pay attention to those with moderate perceived health status. One’s perceived health status is closely related to not only one’s comprehensive health status, but also to quality of life, and is a tool that can predict medical use or mortality by reflecting health coping ability and social resources [43]. Therefore, in order to reduce unmet healthcare needs, it is necessary to identify individuals’ perceived health status and to establish a system and policy that enables medical services to be used when necessary.

In this study, predisposing factors and enabling factors were not identified as significant variables. This is contrary to previous studies showing that predisposing and enabling factors such as age, education, occupation, and type of medical coverage influenced unmet healthcare needs [18,20]. The number of samples in this study was 388, which is relatively small compared to studies of 10,000 or more, and the number of variables identified for each factor was relatively small. Although the weighting was considered, the number of respondents was small; hence, further research on the factors influencing unmet healthcare needs considering various variables should include an expanded number of low-income women who are overweight and obese.

This study data may have been affected by various biases due to the fact that self-reported data were analyzed, not data from medical records. Written responses on respondents’ experiences over the past year may have also been affected by recall bias. Despite these limitations, this study makes a significant contribution by confirming the degree of unmet healthcare needs among low-income women who are overweight and obese, identifying the factors that affect the likelihood of unmet healthcare needs, and obtaining basic data for improving satisfaction with healthcare and promoting health in this population. Based on this study, it will be necessary to increase health equity by promoting personal and socioeconomic health policies that especially consider need factors.

This study investigated the characteristics of low-income women who were overweight and obese and their degree of unmet healthcare needs and analyzed differences in unmet healthcare needs according to respondents’ characteristics and the factors influencing unmet healthcare needs using Anderson’s model of medical use behavior. It was found that 19.4% of low-income women who were overweight and obese experienced unmet healthcare needs and perceived health status had an effect on unmet healthcare needs. Healthcare providers need strategies to develop policies that focusing on the physical and psychological health status of low-income overweight or obese women to reduce unmet healthcare needs.

## Figures and Tables

**Table 1.** General characteristics of respondents (N=388)

Factor	Variable	Categories	n^†^	% ^†^(SE)
Predisposing factors	Age (year)		Mean ± SE, 66.51 ± 1.05	
		<60	68	23.5 (3.2)
		60–69	106	24.7 (2.7)
		≥70	214	51.9 (2.9)
	Marital status	Married	182	47.7 (3.0)
		Bereaved	160	38.6 (2.8)
		Divorced	35	9.4 (1.6)
		Single	11	4.4 (1.5)
	Education level	≤Elementary school	282	67.8 (3.2)
		Middle school	39	10.3 (1.6)
		High school	50	17.1 (2.8)
		≥College	17	4.8 (1.2)
	Family members in the residence (n)		Mean ± SE, 2.04 ± 0.07	
		Alone	160	33.5 (2.9)
		≥2	228	66.5 (2.9)
Enabling factors	Employment	Yes	120	32.3 (3.0)
		No	268	67.7 (3.0)
	Health insurance	Self-employed	117	33.2 (3.0)
		Employee	193	49.4 (3.1)
		Dependent	78	17.4 (2.7)
	Private insurance	Yes	145	40.9 (2.8)
		No	243	59.1 (2.8)
Need factors	Chronic disease			
	Hypertension	Yes	229	52.2 (2.9)
		No	159	47.8 (2.9)
	Diabetes mellitus	Yes	89	21.5 (2.4)
		No	299	78.5 (2.4)
	Tuberculosis	Yes	15	3.5 (0.9)
		No	373	96.5 (0.9)
	Cancer	Yes	33	8.3 (1.6)
		No	355	91.7 (1.6)
	Aerobic physical activity	Yes	87	24.4 (2.7)
		No	301	75.6 (2.7)
	Depression	Yes	119	30.4 (2.9)
		No	269	69.6 (2.9)
	Stress	Yes	127	34.3 (2.5)
		No	261	65.7 (2.5)
	Perceived health status	Poor	180	42.9 (3.1)
		Moderate	176	49.2 (3.1)
		Good	32	7.9 (1.5)

† Unweighted count (frequency), weighted %.

**Table 2.** Unmet healthcare needs in respondents (N=388)

Variable	Categories	n^†^	%^†^ (SE)
Unmet medical needs	Yes	74	19.4 (2.6)
	No	314	80.6 (2.6)

† Unweighted count (frequency), weighted %.

**Table 3.** Unmet healthcare needs according to general characteristics (N=388)

Factor	Variable	Categories	Unmet healthcare needs		F^‡^ or t (*p*)
			n^†^/% (SE)		
			Yes	No	
Predisposing factors	Age (year)		Mean±SE, 68.29±1.33	Mean±SE, 66.08±1.25	–1.20(.232)
		<60	14/17.6 (5.1)	54/82.4 (5.1)	0.96(.385)
		60–69	26/25.8(6.3)	80/74.2 (6.3)	
		≥70	34/17.2 (3.2)	180/82.8 (3.2)	
	Marital status	Married	38/21.4 (3.9)	144/78.6(3.9)	1.17(.319)
		Bereaved	30/20.7 (4.2)	130/79.3 (4.2)	
		Divorced	4/8.7 (4.9)	31/91.3 (4.9)	
		Single	2/9.5 (7.3)	9/90.5 (7.3)	
	Education level	≤ Elementary school	57/22.5 (3.1)	225/77.5 (3.1)	1.94(.131)
		Middle school	6/14.8 (6.1)	33/85.2 (6.1)	
		High school	9/13.4 (4.6)	41/86.6 (4.6)	
		≥College	2/6.9 (4.9)	15/93.1 (6.9)	
	Number of family members in the residence		Mean±SE, 2.33±0.23	Mean±SE, 1.97±0.07	–1.47(.144)
		Alone	30/18.7 (3.7)	130/81.3 (3.7)	0.05(.824)
		≥2	44/19.8 (3.4)	184/80.2 (3.4)	
Enabling factors	Employment	Yes	21/18.2 (4.1)	99/81.8 (4.1)	0.16(.687)
		No	53/20.0 (3.0)	215/80.0 (3.0)	
	Health insurance	Self-employed	22/21.2 (4.6)	95/78.8 (4.6)	0.21(.811)
		Employee	34/18.3 (3.0)	159/81.7 (3.0)	
		Dependents	18/19.4 (47)	60/80.6 (4.7)	
	Private insurance	Yes	28/20.6 (4.5)	117/79.4 (4.5)	0.15(.701)
		No	46/18.6 (3.0)	197/81.4 (3.0)	
Need factors	Chronic disease				
	Hypertension	Yes	43/20.1 (3.1)	186/79.9 (3.1)	0.09(.760)
		No	31/18.7 (3.7)	128/81.3 (3.7)	
	Diabetes mellitus	Yes	12/11.9 (3.6)	77/88.1 (3.6)	3.44(.066)
		No	62/21.5 (3.2)	237/78.5 (3.2)	
	Tuberculosis	Yes	6/34.4 (12.3)	9/65.6 (12.3)	2.06(.155)
		No	68/18.9 (2.7)	305/81.1 (2.7)	
	Cancer	Yes	5/13.3 (5.9)	28/86.7 (5.9)	0.83(.344)
		No	69/20.0 (2.8)	286/80.0 (2.8)	
	Aerobic physical activity	Yes	18/18.5 (4.6)	69/81.5 (4.6)	0.06(.803)
		No	56/19.7 (2.9)	245/80.3 (2.9)	
	Depression	Yes	33/26.3 (4.8)	86/73.7 (4.8)	4.27(.041)
		No	41/16.4 (2.8)	228/ 83.6 (2.8)	
	Stress	Yes	34/26.0 (4.4)	93/74.0 (4.4)	5.93(.017)
		No	40/16.0 (2.6)	221/84.0 (2.6)	
	Perceived health status	Poor	49/28.7 (3.9)	131/71.3 (3.9)	6.92(.001)
		Moderate	21/12.6 (3.2)	155/87.4 (3.2)	
		Good	4/11.4 (5.6)	28/88.6 (5.6)	

† Unweighted count (frequency), weighted %;

‡ Rao-Scott composite sample chi-square tests.

**Table 4.** Factors related to unmet healthcare needs (N=388)

Factor	Variable	Categories	OR (95% CI)	*p*
Need factors	Depression	Yes	1.17 (0.50–2.72)	.718
		No	Reference	
	Stress	Yes	1.40 (0.66–3.01)	.379
		No	Reference	
	Self-related health status	Good	0.37 (0.11–1.27)	.114
		Moderate	0.40 (0.20–0.78)	.007
		Poor	Reference	
χ^2^=15.47, *p*=.003				

CI: Confidence interval; OR: odds ratio.
